# Fast MR Simulation Using Combined Update of Grouped Isochromats

**DOI:** 10.2463/mrms.mp.2025-0131

**Published:** 2026-02-28

**Authors:** Hidenori Takeshima

**Affiliations:** Advanced Technology Research Department, Research and Development Center, Canon Medical Systems Corporation, Tokyo, Japan

**Keywords:** Bloch equations, combined transitions, grouped isochromats, magnetic resonance simulation

## Abstract

**Purpose:**

This work aims to overcome an assumption of conventional MR simulators: Individual isochromats should be simulated individually.

**Methods:**

To reduce the computational times of MR simulation, a new simulation method using grouped isochromats is proposed. When multiple isochromats are grouped before simulations, some parts of the simulation can be shared in each group. For a certain gradient type, the isochromats in the group can be easily chosen for ensuring that they behave the same. For example, the group can be defined as the isochromats whose locations along x-axis, T1, T2 and magnetic field inhomogeneity values are the same values. In such groups, simulations can be combined when a pulse sequence with the magnetic field gradient along x-axis only are processed. The processing times of the conventional and proposed methods were evaluated with several sequences including echo-planar imaging (EPI) and spiral sequences.

**Results:**

The simulation times of the proposed method were 1.7 to 161 times faster than those of the conventional methods. In the cases of 2.3 billion isochromats using single instruction, multiple data (SIMD) instructions and multi-threading, the conventional method simulated the EPI and spiral sequences in 5526.7 and 15678.4 seconds, respectively. In the same cases, the proposed method simulated these sequences in 652.2 and 8046.2 seconds, respectively.

**Conclusion:**

The proposed method using grouped isochromats was efficient for reducing the computational times of the simulations.

## Introduction

MR simulation is an important part of the development, prototyping, optimization, and evaluation of various aspects of MR. There are many implementations^1^^–^^13^ for simulating the Bloch equations and their extensions.^14^^–^^16^ In MR simulators, the magnetizations of isochromats are updated using small step sizes in the order of microseconds. Realistic simulations require millions of isochromats. Even when the spatial resolution of the phantom is not high, it is known that the number of subvoxels should be increased for suppressing artifacts.^9^

A widespread problem of the simulations was to reduce the computational times. Existing work used algorithm-based and hardware-based methods for reducing the computational times. The algorithm-based methods reduced the amount of computation. These methods included utilization of combined transitions,^1^^,^^2^^,^^11^^,^^13^ and no temporal changes.^7^ The hardware-based methods used parallel computing. These methods included utilization of multi-threading,^6^^,^^9^^,^^13^ single instruction, multiple data (SIMD),^9^^,^^13^ general-purpose graphics processing unit (GPGPU),^7^^–^^9^^,^^12^ computer clusters,^3^^,^^6^ and cloud computing.^10^ These methods could reduce the computational times. However, the computational costs of the MR simulation were still high when millions of isochromats were used.

The aim of this paper is to accelerate the simulations by overcoming a common assumption of conventional MR simulators: Individual isochromats should be simulated individually. Under this assumption, isochromats could be updated simultaneously only if they were computed in parallel using the hardware-based methods. In several practical cases such as developing and prototyping software of MR system, acquisition, and reconstruction, it is useful to simulate pulse sequences without relying on such hardware. By utilizing hardware-independent algorithms, it is expected that both simulators with and without the hardware-based methods (including simulators with multi-threading, SIMD, GPGPU, computer clusters, and cloud computing) are accelerated.

To further accelerate the simulations, a new computation method using grouped isochromats is proposed. When multiple isochromats are grouped before a simulation, some parts of the simulation can be shared in each group. For a certain gradient type, the isochromats in the group can be easily chosen for ensuring that they behave the same. In such gradient types, the multiple isochromats were combined and updated simultaneously without relying on the hardware-based methods. The preliminary version of this work was presented as an abstract.^17^

## Materials and Methods

### Basics of MR simulation

Let the direction of the static magnetic field be the z axis. Let the remaining axes representing the rotating frame around the z axis be the x and y axes. Let the time be t. Let the constant γ be the gyromagnetic ratio. In the cases of the Bloch equations,^14^ the magnetization vector $M\left(k,t\right)={\left(M_{x}\left(k,t\right),M_{y}\left(k,t\right),M_{z}\left(k,t\right)\right)}^{T}$ of the k-th isochromat $\left(k=1,\ldots ,K\right)$ satisfies

(1) $$\begin{array}{cc} \frac{dM(k,t)}{dt} & =\gamma \left(M(k,t)\times B(k,t)\right)\\ & +C(k)\left(M_{static}(k)-M(k,t)\right) \end{array}$$

where the static magnetization vector $M_{static}\left(k\right)={\left(0,0,M_{0}\left(k\right)\right)}^{T}$ and the matrix $C\left(k\right)=diag\left(-1/T_{2}\left(k\right),-1/T_{2}\left(k\right),-1/T_{1}\left(k\right)\right)$ represent the constants of the k-th isochromat, and the magnetic field vector $B\left(k,t\right)={\left(B_{x}\left(k,t\right),B_{y}\left(k,t\right),B_{z}\left(k,t\right)\right)}^{T}$ represents a pulse sequence to be simulated at the k-th isochromat. In the pulse sequence, $B_{1}\left(k,t\right)=B_{x}\left(k,t\right)+iB_{y}\left(k,t\right)$ represents the RF pulses, $B_{z}\left(k,t\right)=G\left(t\right)r\left(k,t\right)+\Delta B_{0}\left(k\right)$ represents the magnetic fields from the gradient fields $G\left(t\right)={\left(G_{x}\left(t\right),G_{y}\left(t\right),G_{z}\left(t\right)\right)}^{T}$ at the k-th location $r\left(k,t\right)={\left(r_{x}\left(k,t\right),r_{y}\left(k,t\right),r_{z}\left(k,t\right)\right)}^{T}$ and inhomogeneity $\Delta B_{0}\left(k\right)$. In the cases of static isochromats, $r\left(k,t\right)$ is independent of time and thus is simplified as $r\left(k\right)={\left(r_{x}\left(k\right),r_{y}\left(k\right),r_{z}\left(k\right)\right)}^{T}$.

**Fig. 1:**
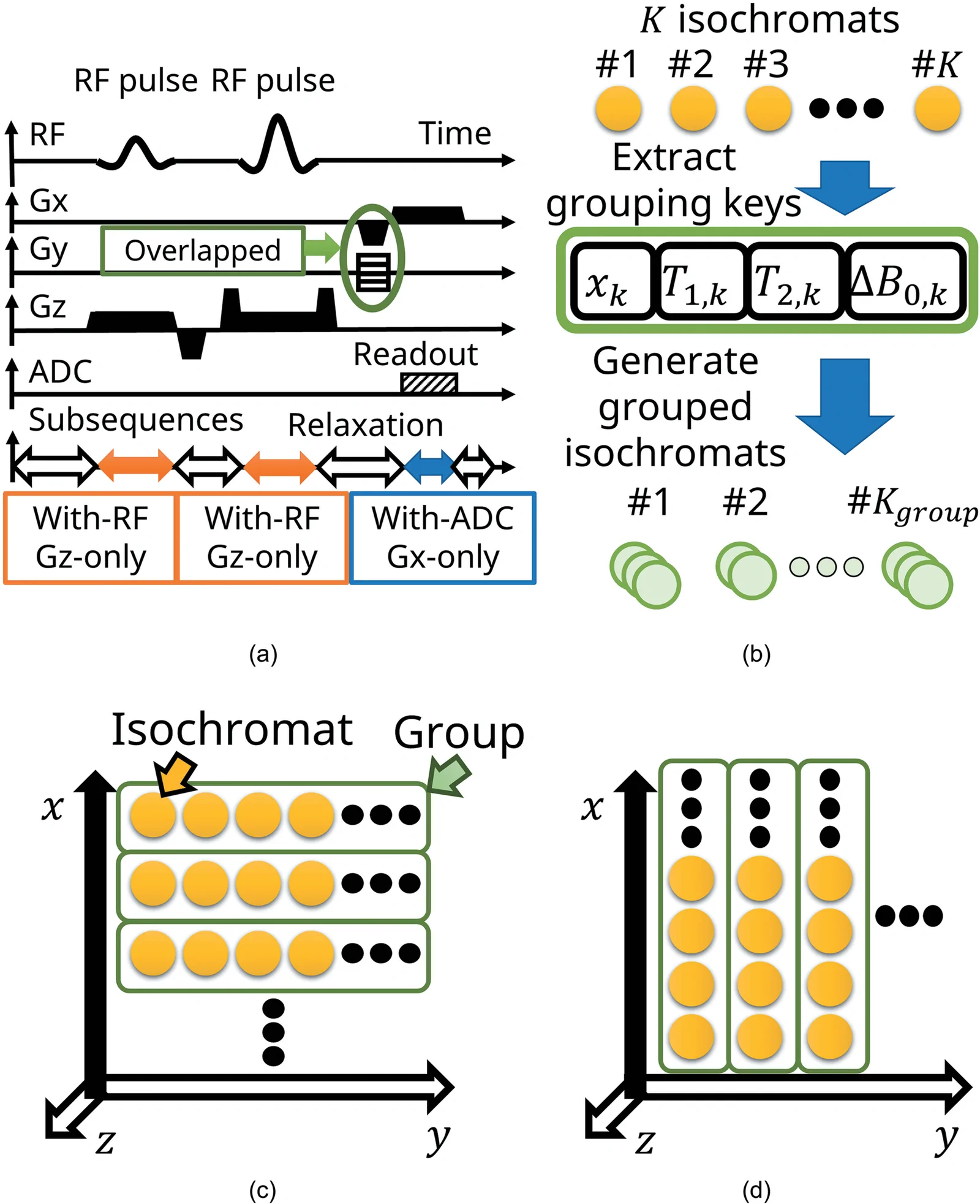
(**a**) Types of subsequences and their groups. The durations where RF pulses are active are classified as with-RF subsequences. The duration where ADC is active is classified as a with-ADC subsequence. Remaining durations are classified as relaxation subsequences. (**b**) A process flow for generating grouped isochromats. For each isochromat, values which should be shared are extracted as the grouping key. The isochromats whose grouping keys are same are grouped. (**c**) An example case for updating magnetizations efficiently in the cases of Gx-only. The isochromats in a group share their *r_x_(k,t), T*_1_*(k), T*_2_*(k)* and Δ*B*_0_ values. (**d**) Another example case for updating magnetizations efficiently in the cases of Gy-only. The isochromats in a group share their *r_y_(k,t), T*_1_*(k), T*_2_*(k)* and Δ*B*_0_ values.ADC, analog-to-digital converter.

This work treated a pulse sequence as a series of subsequences. As shown in Fig. 1, the subsequences can be classified into 3 types: (a) a with-RF subsequence which includes an RF pulse, (b) a with-ADC subsequence which contains a sampling time using analog to digital converters (ADCs), and (c) a relaxation subsequence which contains no RF pulses and sampling times. Subsequences with both an RF pulse and a sampling time simultaneously are not commonly used and thus are not considered.

When general subsequences are processed, iterative update is required for computing the magnetization. For a small duration Δt, the transition of the magnetization vector $M\left(k,t\right)$ between t and t+Δt can be expressed as

(2) $$M\left(k,t+\Delta t\right)=U\left(k,t,\Delta t\right)M\left(k,t\right)$$

where $U\left(k,t,\Delta t\right)$ is a 4×4 matrix representation of the transition given in Appendix A, and $M\left(k,t\right)=\;{\left(M_{x}(k,t),M_{y}(k,t),M_{z}(k,t),M_{0}(k)\right)}^{T}$ contains $M\left(k,t\right)$ and the non-zero element of $M_{static}\left(k\right)$. The magnetic field vector $B\left(t\right)$ is assumed to be piecewise constant between t and t+Δt. By combining Eq. (2) at $t=t_{0},t_{1},\ldots ,t_{N_{RF}-1}$, a combined transition between $t_{0}$ and $t_{N_{RF}}$ can be expressed as

(3) $$\begin{array}{cc} M\left(k,t_{N_{RF}}\right) & =U\left(k,t_{N_{RF}-1},\Delta t_{N_{RF}-1}\right)\cdots \\ & U\left(k,t_{1},\Delta t_{1}\right)U\left(k,t_{0},\Delta t_{0}\right)M\left(k,t_{0}\right) \end{array}$$

if all durations $\Delta t_{j}=t_{j+1}-t_{j}$ ($j=0,\ldots ,N_{RF}-1$) are sufficiently small.

In the cases of the special subsequences which satisfy $B_{1}\left(k,t\right)=0$ for $t_{0}\leq t\leq t_{1}$, the magnetization at t can be computed using the following analytic solutions

(4) $$\begin{array}{cc} M_{xy}(k,t) & M_{xy}(k,t_{0})\exp\left(-\frac{t-t_{0}}{T_{2}(k)}\right)\\ & \exp\left(\int_{t_{0}}^{t}-i\gamma B_{z}(k,t)dt\right),\;\text{and} \end{array}$$

(5) $$\;\;\;M_{z}\left(k,t\right)=M_{0}\left(k\right)+(M_{z}\left(k,t_{0}\right)-M_{0}\left(k\right))\exp\left(-\frac{t-t_{0}}{\frac{}{}}T_{1}\left(k\right)\right)$$

if the k-th isochromat is static. These solutions can be applied to the with-ADC and relaxation subsequences.

In the with-ADC subsequences, k-space data sampled at the time t are represented as

(6) $$A\left(p,t\right)=\overset{K}{\underset{k=1}{\sum }}S\left(k,p,t\right)M_{xy}\left(k,t\right)$$

where $S\left(k,p,t\right)$ represents the sensitivity of the p-th receiver-coil (p=1,2,…,P), and $M_{xy}\left(k,t\right)=M_{x}\left(k,t\right)+iM_{y}\left(k,t\right)$ represents the transverse magnetizations.

### Computational complexities of conventional methods

In the cases of the with-RF subsequences, naïve implementations of Eq. (3) have a complexity of $O\left(N_{RF}K\right)$. To accelerate computation of these subsequences, methods based on the combined transitions^1^^,^^2^^,^^11^^,^^13^ can be utilized. These methods split the update process of Eq. (3) into the preparation and update steps.

In the preparation step, the combined transition

(7) $$\begin{array}{r} T\left(k,t_{0},t_{N_{RF}}\right)=U\left(k,t_{N_{RF}-1},\Delta t_{N_{RF}-1}\right)\cdots \\ U\left(k,t_{1},\Delta t_{1}\right)U\left(k,t_{0},\Delta t_{0}\right) \end{array}$$

is computed. The preparation step has a complexity of $O\left(N_{RF}K\right)$. This step can be implemented as either precomputing^1^^,^^2^^,^^11^ or dynamic caching.^13^ Once $T\left(k,t_{0},t_{N_{RF}}\right)$ is computed, the complexity of the update step is reduced to $O\left(K\right)$ since only a single matrix multiplication $M\left(k,t_{N_{RF}}\right)=T\left(k,t_{0},t_{N_{RF}}\right)U\left(k,t_{0},\Delta t_{0}\right)$ is required for all K isochromats. While the complexity of the update step is lower than that of the naïve implementations, the complexity of the preparation step is not changed.

When isochromats are static, the with-ADC and relaxation subsequences have complexities of $O\left(N_{ADC}PK\right)$ and $O\left(K\right)$, respectively. According to the Eqs. (4) and (5), $M\left(k,t\right)$ can be computed directly within $t_{0}\leq t\leq t_{1}$. In the cases of the with-ADC sequences, $N_{ADC}$ represents the number of ADC samples to be acquired with Eq. (6).

The main load of computation is the simulations of the with-RF and with-ADC subsequences. In realistic sequences to be simulated, the typical number of isochromats K is in the order of millions. The typical numbers of RF update steps $N_{RF}$ and ADC samples $N_{ADC}$ are in the range of dozens to thousands.

### Efficient simulations of with-RF and with-ADC subsequences

The computational complexities can be reduced if those of the with-RF and with-ADC subsequences are reduced. This subsection shows that the computational complexities of simulations can be reduced under the following assumption: When a subsequence is simulated, there are several groups where multiple isochromats share $U\left(k,t,\Delta t\right)$. This assumption means that the underlying components $B_{1}\left(k,t\right)$, $B_{z}\left(k,t\right)$, $T_{1}\left(k\right)$ and $T_{2}\left(k\right)$ are all shared.

In the cases of the with-RF subsequences under this assumption, the preparation step can be implemented as computing $T\left(k,t_{0},t_{N_{RF}}\right)$ once in each group. The combined transition can be used in the update step of all isochromats in the group. Therefore, the complexity of the preparation step can be reduced from $O\left(N_{RF}K\right)$ to $O\left(N_{RF}K_{group}\right)$ where $K_{group}$ represents the number of the groups. The complexity of the update step is still $O\left(K\right)$ since the same update step is used.

In the cases of the with-ADC subsequences under this assumption, Eq. (6) can be rewritten as

(8) $$A\left(p,t\right)=\overset{K_{group}}{\underset{k_{group}=1}{\sum }}A_{block}\left(k_{group},p,t\right)$$

where $A_{block}\left(k_{group},p,t\right)$ is defined as

(9) $$A_{block}(k_{group},p,t)=\sum_{k\in L(k_{group})}S(k,p,t)M_{xy}(k,t)$$

and $L\left(k_{group}\right)$ represents a set of isochromats in the $k_{group}$-th group. By assigning Eq. (4) into Eq. (9), $A_{block}\left(k_{group},\;p,t\right)$ for $t_{0}\leq t\leq t_{1}$ can be represented as

(10) $$\begin{array}{c} A_{block}\left(k_{group},p,t\right)=\exp\left(-\frac{t-t_{0}}{T_{2}(k_{group})}\right)\;\text{.}\\ \exp\left(\int_{t_{0}}^{t}-i\gamma B_{z}\left(k_{group},t\right)dt\;\right)A_{block}\left(k_{group},p,t_{0}\right) \end{array}$$

where $T_{2}\left(k_{group}\right)$ and $B_{z}\left(k_{group},t\right)$ represent shared components in the $k_{group}$-th group.

By utilizing Eq. (10), the with-ADC subsequences can be simulated using the following 3-step method. As the first step whose time is $t_{0}$, compute $A_{block}\left(k_{group},p,t_{0}\right)$ using Eq. (9). This step reduces K isochromats into $PK_{group}$ groups. As the second step, compute $N_{ADC}$ sampling values using Eqs. (8) and (10) for $PK_{group}$ groups. As the third step, update $M\left(k,t\right)$ using Eqs. (4) and (5) for K isochromats. The first, second, and third steps have complexities of $O\left(PK\right)$, $O\left(N_{ADC}PK_{group}\right)$ and $O\left(K\right)$, respectively.

### Grouping criteria for efficient simulations

This subsection shows simple criteria for grouping isochromats. It is assumed that the grouping process of the isochromats is performed once. Unless the computational times of the grouping operation is too great, the simulations can be accelerated if types of subsequences can be identified with trivial computational times.

This work considers special gradient types found in common pulse sequences: Gx-only, Gy-only, Gz-only, and no-grads. These criteria assume that the with-RF and with-ADC subsequences tend to have only one active component of $G\left(t\right)$. As shown in Fig. 1a, when the gradient field $G_{x}\left(t\right)$ is non-zero, the remaining gradient fields $G_{y}\left(t\right)$ and $G_{z}\left(t\right)$ are sometimes zeros in the subsequences to be simulated. Under this gradient type, isochromats can be grouped when $r_{x}\left(k,t\right)$, $T_{1}\left(k\right)$, $T_{2}\left(k\right)$ and $\Delta B_{0}\left(k\right)$ values are the same values, as shown in Fig. 1b–d. This gradient type is referred to as Gx-only. Similar gradient types with single non-zero gradient fields of $G_{y}\left(t\right)$ and $G_{z}\left(t\right)$ can be defined in the cases of Gy-only and Gz-only, respectively. The no-grads type represents the subsequences whose gradient fields are zeros. Its example cases are chemical shift selective (CHESS) pulses^18^ and sequences for adjusting RF power levels.^19^^,^^20^

The difference between the proposed and conventional methods is the strategy for updating multiple isochromats simultaneously. The conventional methods accelerated simulations by updating them in parallel with various hardware.^3^^,^^6^^–^^10^^,^^12^^,^^13^ In contrast, the proposed method updates them by utilizing grouped isochromats and thus is applicable to both non-parallel and parallel computing environments.

### Overview of evaluations

To evaluate the performance of MR simulation methods, the proposed method was implemented as a part of house-made software^13^ named a virtual MR scanner (VMRscan). The software has a conventional acceleration method using the combined transitions with a dynamic caching method for simulating the with-RF subsequences. The software was run on a central processing unit (CPU) with 8 performance cores, 16 efficient cores, and 32 processor threads. The frequencies of the CPU were 3.2 GHz for the performance cores and 2.4 GHz for the efficient cores. These cores were dynamically boosted up to 6.0 GHz.

In addition to the simulation method, evaluations require phantoms and sequences. As phantoms, numeric and measured phantoms were developed. The numeric phantom was the one used in the previous work.^13^ The measured phantom was generated with a simple parameter mapping method and preprocessed with a clustering algorithm. For the sake of evaluations, 4 pulse sequences distributed as a part of the Pulseq version 1.5.0^21^ were used.

The efficiency was evaluated using the VMRscan with and without the proposed method. The efficiency was also evaluated with and without an artificial $\Delta B_{0}$ maps.

### Fast simulations with grouped isochromats

**Fig. 2:**
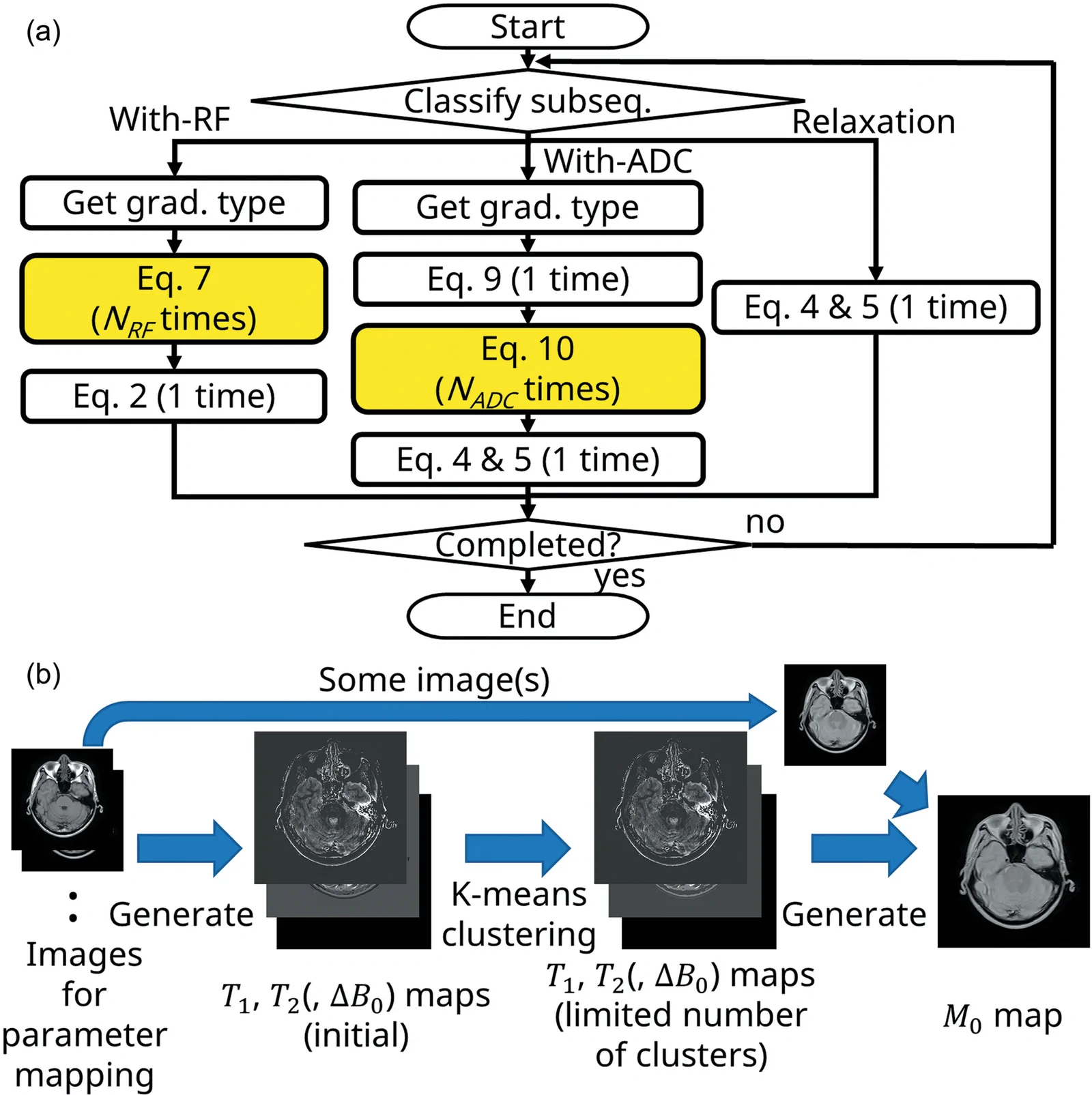
(**a**) Overview of the proposed method. Each subsequence is classified into 3 types. In the cases of with-RF and with-ADC subsequences, grouped isochromats are used in time-consuming processes. When the grouping criteria are not met in a subsequence, the subsequence is simulated using conventional methods (not shown in this figure). (**b**) Preprocessing method for ensuring that the numbers of groups are sufficiently small. For reducing the number of groups, a clustering algorithm was applied to the initial maps consisting of *T*_1_*, T*_2_ and Δ*B*_0_ maps. The remaining map *M*_0_ was generated using the clustered parameters and existing images used in the parameter mapping of the initial parameter maps.ADC, analog-to-digital converter.

The proposed method split a sequence into a series of subsequences, classified the subsequence individually, and simulates the subsequences using one of 3 different flows as shown in Fig. 2a. In the cases of the with-RF and with-ADC subsequences, gradient types of the subsequences were classified before simulating the subsequences. Whenever gradient types of subsequences were classified as one of known types, grouped simulations explained in the theory were utilized. Remaining subsequences were simulated with a conventional method.^13^

The relaxation subsequences could be processed quickly regardless of whether these subsequences had specific gradient types or not. The accelerations using specific gradient types were applied to the with-RF and with-ADC subsequences only. It is worth noting that practical pulse sequences often used overlapped gradients for reducing TE as shown in Fig. 1. Such overlapped gradients do not affect the performance of the proposed method which accelerates the with-RF and with-ADC subsequences since the overlapped gradients are commonly used in relaxation subsequences.

### Numeric and measured phantoms

**Fig. 3:**
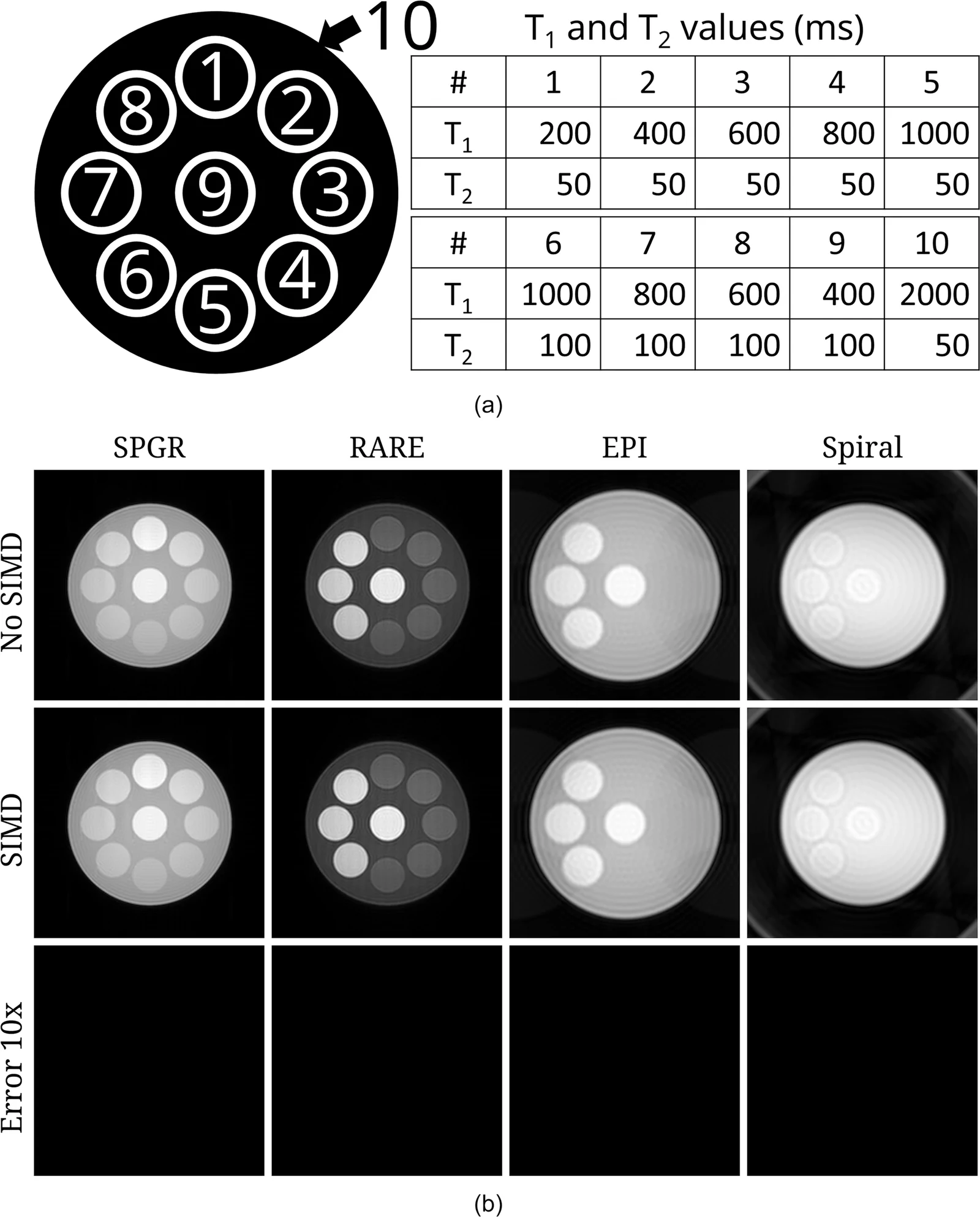
(**a**) The circles phantom. Each circle has a unique pair of *T*_1_ and *T*_2_ values. (**b**) Reconstructed images and errors using the circles phantom and the proposed method. Four sequences were used for the simulations. There were no visible differences between reconstructed images with and without the proposed method since the differences were the errors caused by floating-point computations only. The images using the spiral sequence included artifacts generated by the gridding algorithm at the corners.EPI, echo-planar imaging; RARE, rapid acquisition with relaxation enhancement; SIMD, single instruction, multiple data; SPGR, spoiled gradient echo.

A numeric phantom named circles was used as a phantom suitable for the proposed method. The phantom contained a large cylinder and 9 small cylinders. These small cylinders were put in the large cylinder. Its 2D planes consisted of 10 circles as shown in Fig. 3a. The matrix and spatial sizes of the phantom were 960×960×48 and 240 mm×240 mm×12 mm, respectively. In the phantom, 20068128 isochromats (1 isochromat per pixel) were put within the large circle. In each cylinder, $T_{1}\left(k\right)$ and $T_{2}\left(k\right)$ values were constants as shown in Fig. 3a. All $\Delta B_{0}\left(k\right)$ values were set to 0 Hz. Number of groups were 10, 2108, 2114, and 480 for no-grads, Gx-only, Gy-only, and Gz-only, respectively. This phantom is suitable for simulating pulse sequences with small numbers of groups in all gradient types.

A measured phantom named brain was generated from multi-slice images of a brain. These images were acquired in a volunteer scan. The pulse sequences used in the scan are shown in Table 1. These images consisted of 4 T1-weighted (T1W) images and 4 T2-weighted (T2W) images. All acquisitions shared the matrix and FOV sizes. The number of readouts, phase encodes, and slices were 256, 160 and 48, respectively. The size of reconstructed images was 256×240×48 by zero-filling unknown k-space data for scaling the images in the readout and phase encoding directions. The FOV size was 256 mm×240 mm×240 mm. The $T_{1}\left(k\right)$ and $T_{2}\left(k\right)$ values were estimated using the following parameter mapping method. For each pixel in the $T_{1}$ and $T_{2}$ maps, the parameter mapping method searched the best value from a set of discrete candidate values. The candidate values were put in the range of 10 to 3000 milliseconds for the $T_{1}$ map, and 10 to 500 milliseconds for the $T_{2}$ map. For individual maps, 100 logarithmically spaced candidate values were used. The $M_{0}\left(k\right)$ values were estimated using the T2W images, $T_{1}$ map and $T_{2}$ map. Any voxels whose $M_{0}\left(k\right)$ values were lower than a threshold were ignored. There were 2 types of the $\Delta B_{0}\left(k\right)$ values: with and without an artificial $\Delta B_{0}$ map. The artificial $\Delta B_{0}$ map was defined as $\Delta B_{0}\propto \left(x/FOV_{x}+\;y/FOV_{y}+z/FOV_{z}\right)$ where $\left(FOV_{x},FOV_{y},FOV_{z}\right)$ represented FOV of the brain phantom. The minimum and maximum values of the artificial $\Delta B_{0}$ map were scaled to −200 and 200 Hz. When the artificial $\Delta B_{0}$ map was not used, all $\Delta B_{0}\left(k\right)$ values were set to 0 Hz. The volunteer scan was approved by the institutional review board of Canon Medical Systems Corporation, and informed consent was obtained from the volunteer.

### Preprocessing method for grouping isochromats efficiently

When the number of groups is great, the proposed method cannot reduce computational time of simulations. In the cases of numerical phantoms such as the circles phantom, all numbers of groups using above-mentioned criteria can be controlled by design. However, in general cases, the numbers of groups are close to the number of isochromats. To use the proposed method efficiently, phantoms could be preprocessed if it is acceptable to change time constants and field inhomogeneity of isochromats slightly.

To ensure that the numbers of groups are sufficiently small, phantoms can be preprocessed using an arbitrary clustering algorithm. The overview of the preprocessing method is shown in Fig. 2b. Initial parameter maps can be generated by an arbitrary parameter mapping method. To generate parameter maps with a limited number of parameters, a clustering algorithm was applied to the initial parameter maps. The space of the clustering algorithm was $\left(T_{1},T_{2},\Delta B_{0}\right)$. As the clustering algorithm, a bisecting K-means clustering algorithm in the scikit-learn package^22^ was used. The $M_{0}\left(k\right)$ values were refined using the T2W images and the center values of the updated parameter maps. The locations $r\left(k\right)$ were also aligned when the isochromats were put into voxels.

### Pulse sequences

To evaluate the computational times depending on the type of subsequences, 4 pulse sequences distributed as the samples of the Pulseq version 1.5.0^21^ were used with no modifications. The pulse sequences whose names were gre.seq, tse.seq, epi.seq, and spiral.seq were 2D implementations of spoiled gradient echo (SPGR), rapid acquisition with relaxation enhancement (RARE), echo planar imaging (EPI), and spiral sequences, respectively. The number of the with-RF subsequences were 128, 153, 3, and 8 for the SPGR, RARE, EPI, and spiral sequences, respectively. The number of the with-ADC subsequences were 128, 128, 192, and 4 for the SPGR, RARE, EPI, and spiral sequences, respectively. In the case of the spiral sequence, the proposed method could not accelerate the with-ADC subsequences since the readout gradients consisted of Gx and Gy. In the other 3 sequences, the proposed method could accelerate both the with-RF and with-ADC subsequences. In these sequences, the minimum values of in-plane resolution and slice thickness were 2 mm and 3 mm, respectively. Therefore, when a spatial resolution of a phantom was set to 0.25 mm×0.25 mm×0.25 mm, the minimum numbers of isochromats in in-plane and slice directions were 8×8 and 24, respectively. The parameters used in these sequences are given in Table 2.

### Simulations #1

To evaluate the efficiency of the proposed method, processing times of the following simulations were measured 6 times using the circle phantom whose spatial resolution was 0.25 mm×0.25 mm×0.25 mm. Since no preprocessing was required for this phantom, simulations with and without the proposed method were evaluated without using the preprocessing method. All simulations were evaluated with and without SIMD instructions since the SIMD instructions could not parallelize lookup table operations (for finding isochromat indices from converting group indices) efficiently. In all the simulations, multi-threading implemented in the previous work were used.^13^ The k-space data were reconstructed using a gridding algorithm.^23^ The oversampling factors of the gridding algorithm were 2.0 for the SPGR, RARE, and spiral sequences; and 4.5 for the EPI sequence. The oversampling factor of the EPI sequence was increased for suppressing aliasing artifacts since the sizes of the phantoms were larger than the FOV of the EPI sequence. In the cases of the EPI and spiral sequences, the first slice was reconstructed.

### Simulations #2

To evaluate the performance of simulations with a phantom which required preprocessing, processing times using the brain phantom with and without the artificial $\Delta B_{0}$ map were also measured. The number of isochromats were increased when the brain phantom with the artificial $\Delta B_{0}$ map was processed.

In the cases of the brain phantom without the artificial $\Delta B_{0}$ map, the phantom was divided into 8 partial phantoms, and each simulation was also divided into the 8 partial simulations with the partial phantoms for reducing amount of memory used in the simulations. Each voxel was divided into 4×4×20 subvoxels for adjusting spatial resolution of the phantom to 0.25 mm×0.25 mm×0.25 mm. The evaluated numbers of clusters were 64 and 256.

Similarly, in the cases of the brain phantom with the artificial $\Delta B_{0}$ map, the phantom was divided into 32 partial phantoms, and each voxel was divided into 8×8×40 subvoxels for adjusting spatial resolution of the phantom to 0.125 mm×0.125 mm×0.125 mm. The evaluated numbers of clusters were 256, 512, 1024, 2048, 4096, 8192, and 16384.

All simulations were evaluated with SIMD instructions. These times were measurements once for each condition. Other conditions were same as those in the evaluations #1.

## Results

### Simulations #1

The number of isochromats and groups are shown in Table 3a. The average processing times are shown in Table 3b. While there were 20 million isochromats, the numbers of groups were less than 10000. When SIMD instructions were not used, the simulation times of the proposed method were 63, 58, 161, and 1.7 times faster than those of the conventional methods in the cases of the SPGR, RARE, EPI, and spiral sequences, respectively. The ratios without SIMD instructions were higher than those with SIMD instructions. When SIMD instructions were used, the simulation times of the proposed method were 11, 10, 27, and 2.0 times faster than those of the conventional methods in the cases of the SPGR, RARE, EPI, and spiral sequences, respectively. Reconstructed images using the circles phantom are shown in Fig. 3b. There were no visible differences between reconstructed images with and without the proposed method since the differences were the errors caused by floating-point computations only.

### Simulations #2

**Fig. 4:**
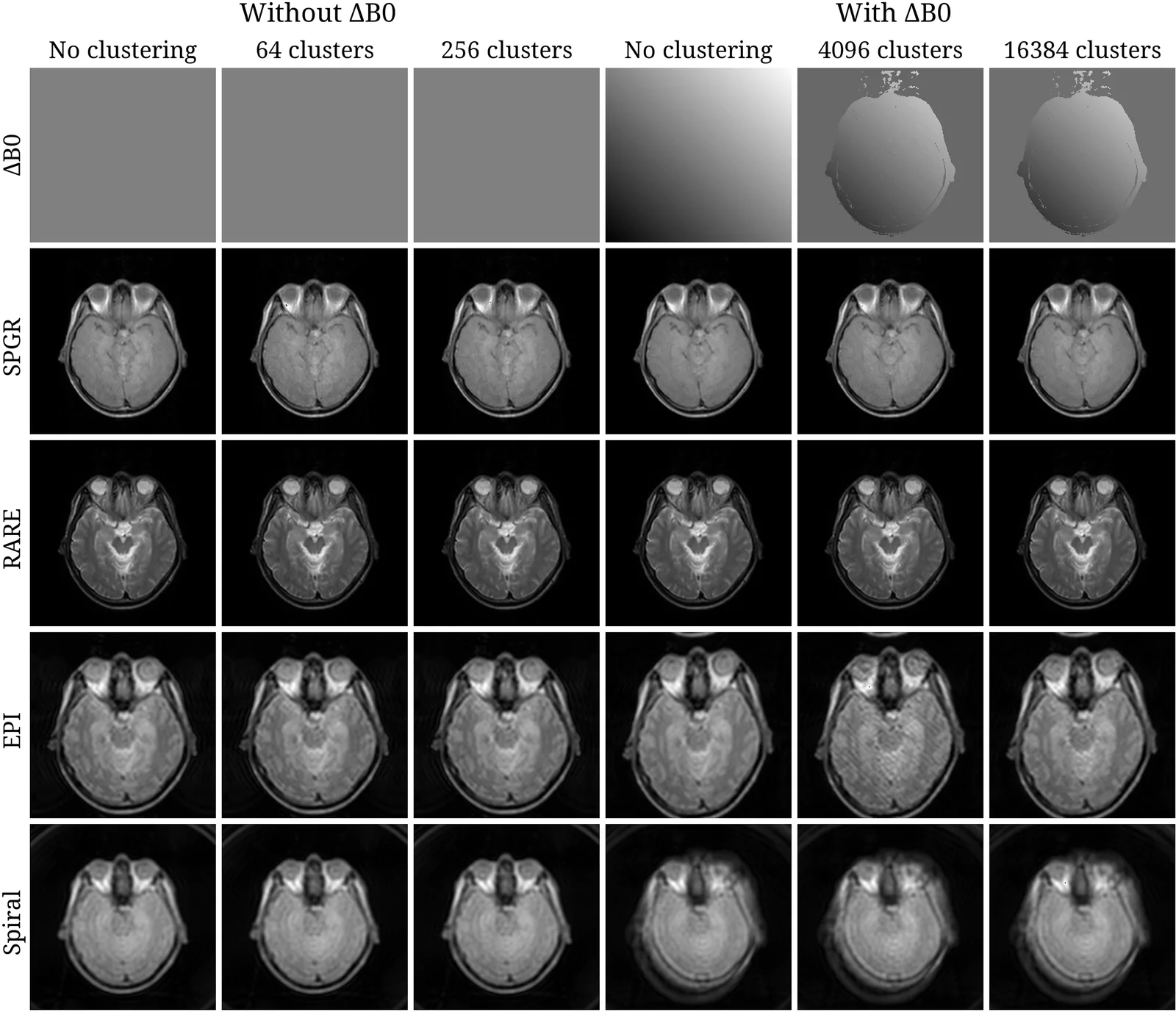
Artificial Δ*B*_0_ map and reconstructed images with and without the artificial Δ*B*_0_ map. Without the artificial Δ*B*_0_ map, the reconstructed images using 256 clusters were visually close to the reference image. When 4096 and 16384 clusters were used with the artificial Δ*B*_0_ map, there were strong and weak artifacts in the reconstructed images of the EPI sequence, respectively. Regardless of whether clustering was used or not, image distortions caused by the artificial Δ*B*_0_ map were found in the results of the EPI and spiral sequences. There were aliasing artifacts in the reconstructed images of the EPI since the FOV of the EPI was less than the size of the brain phantom.EPI, echo-planar imaging; RARE, rapid acquisition with relaxation enhancement; SPGR, spoiled gradient echo.

**Fig. 5:**
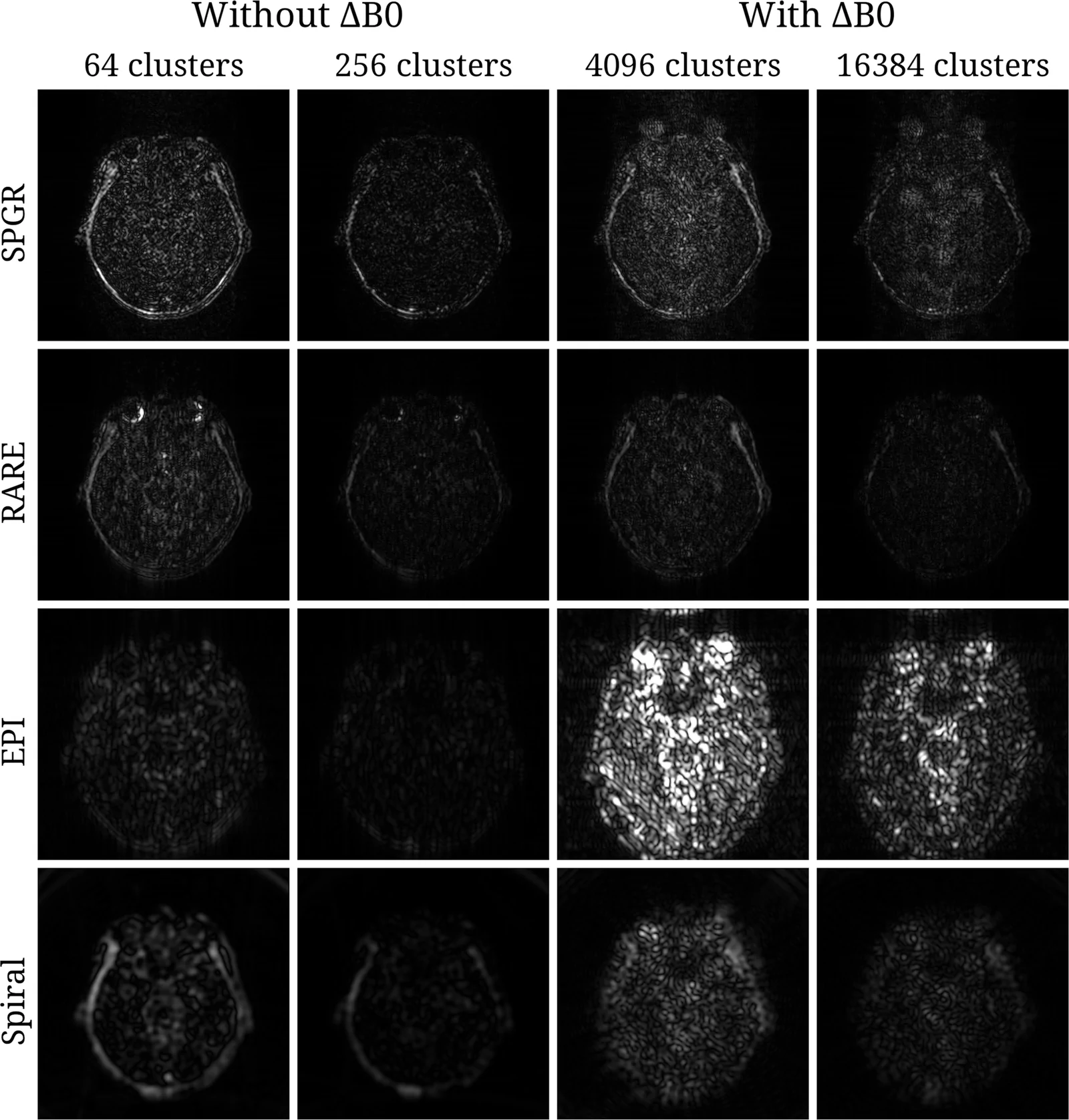
Reconstruction error maps with and without the artificial Δ*B*_0_ map. The errors were multiplied by 10. The errors in the simulated images with the artificial Δ*B*_0_ map were higher than those without the artificial Δ*B*_0_ map. When the artificial Δ*B*_0_ map was used, the errors with SPGR and EPI sequences were higher than those with the RARE and spiral sequences.EPI, echo-planar imaging; RARE, rapid acquisition with relaxation enhancement; SPGR, spoiled gradient echo.

The number of isochromats and mean number of groups are shown in Table 4a. The processing times are shown in Table 4b. While the numbers of isochromats were 284 million and 2.3 billion, the numbers of groups in the partial phantoms were less than 1 million. When the artificial $\Delta B_{0}$ map was used, the conventional method simulated the SPGR, RARE, EPI, and spiral sequences in 5017.3, 5810.0, 5526.7, and 15678.4 seconds, respectively. In the same conditions, the proposed method with 16384 clusters simulated these sequences in 1442.9, 1569.5, 652.2, and 8046.2 seconds, respectively. Therefore, the simulation times of the proposed method were 3.5, 3.7, 8.5, and 1.9 times faster than those of the conventional methods in the cases of the SPGR, RARE, EPI, and spiral sequences, respectively. Representative reconstructed images and error maps are shown in Figs. 4 and 5, respectively. The reconstructed images and error maps which are not shown in Figs. 4 and 5 are shown in Figs. S1 and S2, respectively. Without the artificial $\Delta B_{0}$ map, the reconstructed images using 256 clusters were visually close to the reference image. In the cases of 4096 clusters with the artificial $\Delta B_{0}$ map, there were strong artifacts in the reconstructed images of the EPI sequence. By increasing the number of clusters to 16384 clusters, these artifacts were reduced. In the cases of the SPGR sequence, there were artifacts using the unmodified artificial $\Delta B_{0}$ map and noises using the artificial $\Delta B_{0}$ map modified with clustering. Regardless of whether clustering was used or not, image distortions caused by the artificial $\Delta B_{0}$ map were shown in the reconstructed images of the EPI and spiral sequences. The errors in the simulated images with the artificial $\Delta B_{0}$ map were higher than those without the $\Delta B_{0}$ maps.

**Fig. 6:**
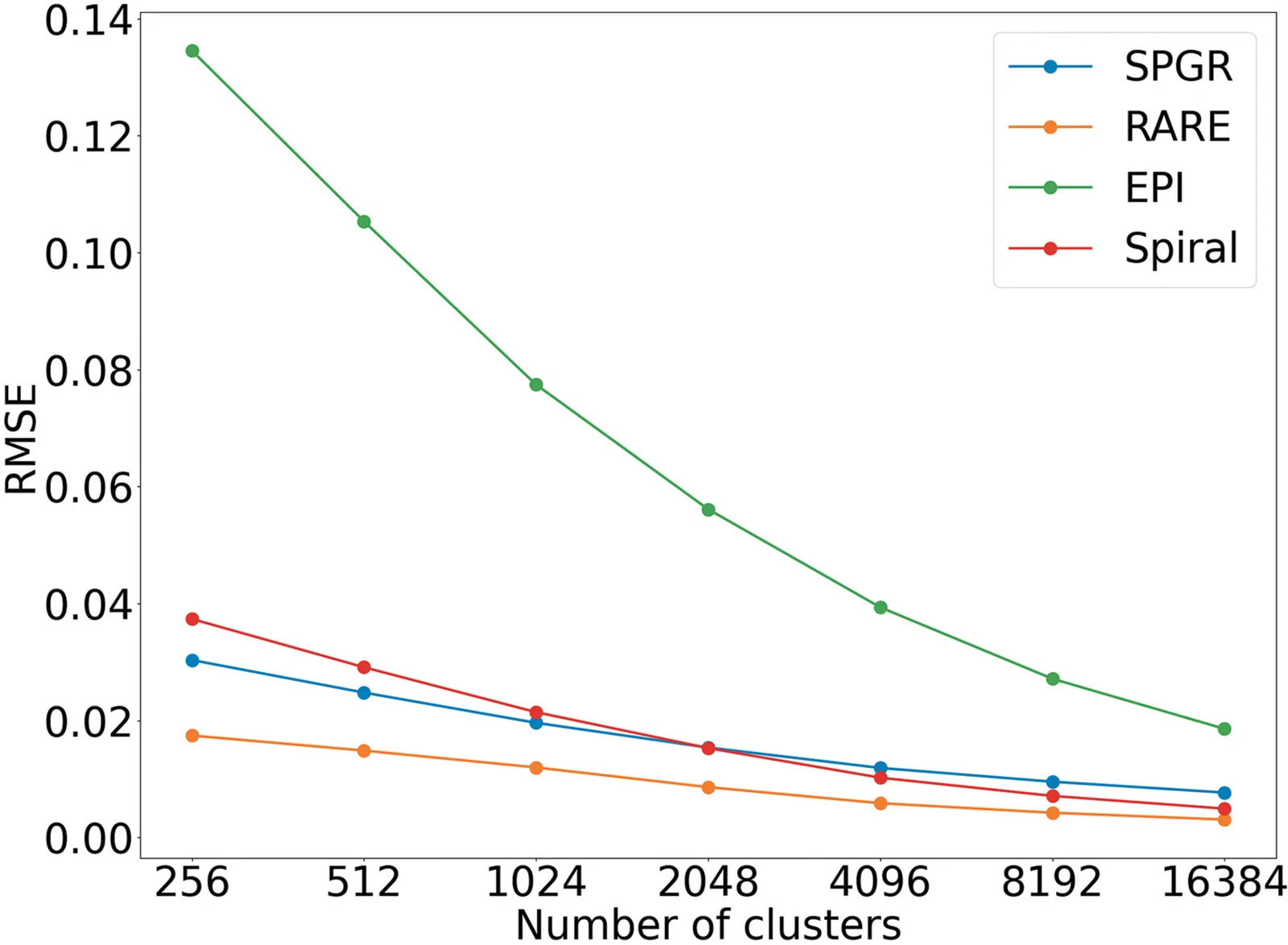
Relationship between numbers of clusters and RMSEs in the cases of the brain phantom with the artificial Δ*B*_0_ map. The lower RMSE is better. The RMSEs of the EPI were higher than other pulse sequences. The legend of the plot represents the sequence name given in Table 2.EPI, echo-planar imaging; RARE, rapid acquisition with relaxation enhancement; RMSE, root mean squared error; SPGR, spoiled gradient echo.

Relationship between numbers of clusters and root mean square errors (RMSEs) in the cases of the brain phantom with the artificial $\Delta B_{0}$ map is shown in Fig. 6. The RMSEs of the EPI were higher than other pulse sequences. The RMSE of the EPI with 16384 clusters (0.0161) was slightly higher than the RMSEs of the SPGR and spiral with 2048 clusters (0.0154 and 0.0153, respectively).

## Discussion

Both evaluations showed that the proposed method further accelerated a conventional acceleration method using the combined transitions.^13^ As shown in Table 3, the proposed method accelerated simulations in the cases of the all tested sequences, including the spiral sequence. In addition, the proposed method was still effective with SIMD instructions even though use of lookup table operations was not effective for parallelization. These results showed that the proposed method efficiently reduced the processing times which included the complexities of $O\left(N_{RF}K\right)$ and $O\left(N_{ADC}PK\right)$. In addition to the efficiency of the proposed method, the differences between Table 3 and Table 4 also showed that the major source of the computational complexity was still the number of isochromats.

The proposed method enlarges suitable environments for simulations. The results of the evaluation #1 also showed that the proposed method reduced the gaps between the processing times with and without special hardware such as SIMD instructions.

The proposed method has a capability of accelerating pulse sequences without using RF pulses repeatedly. Existing methods^1^^,^^2^^,^^11^^,^^13^ could not accelerate such pulse sequences since these methods accelerated simulations by reusing the combined transitions with later subsequences. In contrast, the results of the spiral sequence showed that the proposed method was effective even if only the with-RF subsequences could be accelerated. The results showed that the proposed method was efficient to accelerate the with-RF subsequences by sharing the combined transitions in multiple isochromats.

There is a trade-off between reduction of processing times and loss of faithfulness of measured phantoms. The results showed that errors were decreased gradually when the number of clusters were increased. As shown in Fig. 4, Fig. 5, and Fig. 6, the reconstructed errors depended on both the types of pulse sequences and the numbers of clusters which controlled clustering errors. The clustering errors were reflected to the reconstructed images as artifacts. The quantization errors in the $\Delta B_{0}$ map could cause unintended boundaries such as the artifacts found in the EPI with 4096 clusters. As shown in Table 4, the number of clusters could be increased with a small increase in processing time when both 284 million and 2.3 billion isochromats were used. Since the MR simulators cannot control pulse sequences to be simulated, it is preferred for avoiding the loss of the faithfulness to use moderately large numbers of clusters. If $\Delta B_{0}$ values were constant, the results suggested that 256 clusters were sufficient. When a $\Delta B_{0}$ map was used, increase of the number of isochromats was unavoidable for eliminating artifacts (regardless of whether the proposed method was used or not). In the proposed method with up to 16384 clusters, increase of the number of clusters caused only slight increase of computational times. Therefore, if simulated ranges of field inhomogeneity were less than 400 Hz (approximate 3 ppm at 3T), the number of clusters with 16384 was a reasonable choice for eliminating artifacts caused by $\Delta B_{0}$-sensitive pulse sequences such as the EPI sequence. This trade-off is not a matter in the cases of numeric phantoms.

A limitation of the proposed method is that subsequences which cannot be classified as current gradient types cannot be accelerated. In these subsequences, the number of groups is much increased since 2 or more components in the 3D space must be shared. While it is easy to use additional gradient types, it is not easy to accelerate simulations efficiently with such groups. These subsequences include pulse sequences with eddy current simulations.

## Conclusion

A new computation method using grouped isochromats for accelerating the simulations has been proposed. The proposed method using grouped isochromats was efficient for reducing the computational times of the simulations. Experimental results showed that the simulation times of the proposed method were 1.7 to 161 times faster than those of the conventional methods.

## Figures and Tables

**Figure:**
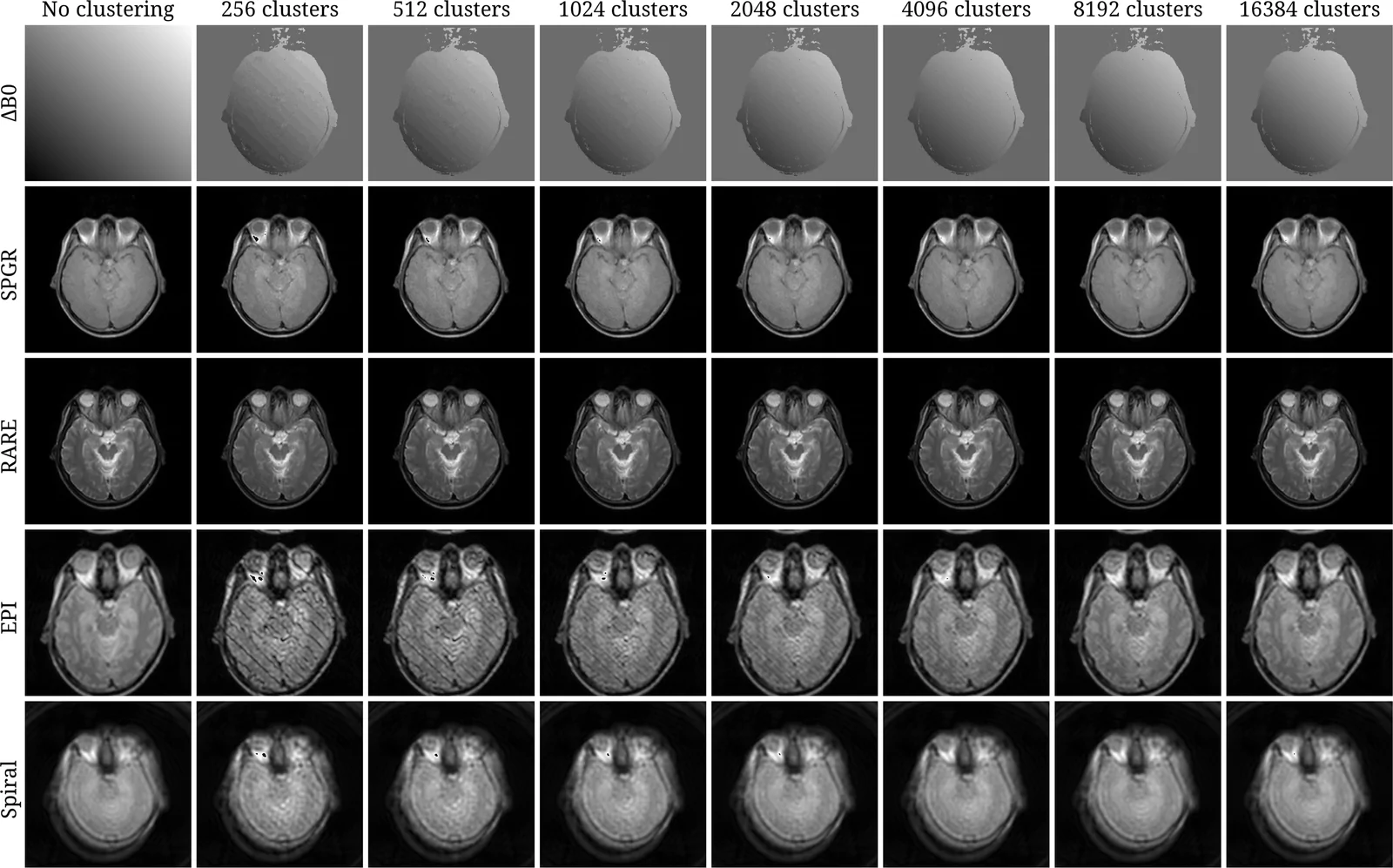


**Figure:**
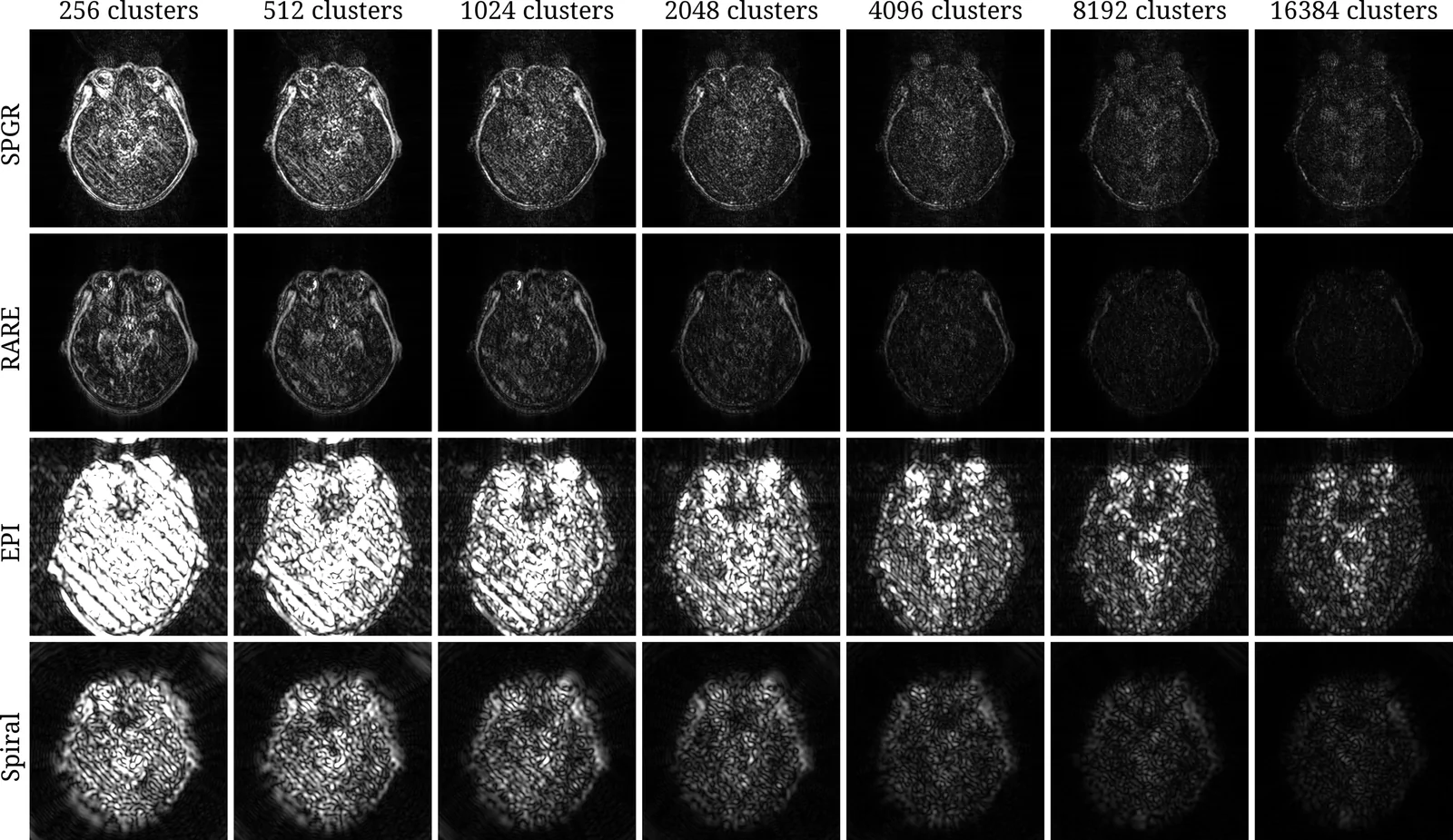


**Table 1:** Pulse sequences used in acquisitions of a volunteer

Pulse sequences used for creating the brain phantom		
	T1W (4 acquisitions)	T2W
Sequence type	2D SE	2D RARE with 4 echos
Acquisition matrix	256 × 160, 48 slices	256 × 160, 48 slices
Reconstruction matrix	512 × 480 × 48	512 × 480 × 48
FOV (mm × mm × mm)	256 × 240 × 240	256 × 240 × 240
Num. echos	1	4
TE (ms)	10	20, 60, 100, 140
TR (ms)	500, 800, 1100, 1500	4500

In the acquisitions, 48 slices were acquired for 8 different image contrasts. The acquired images were used for creating the brain phantom. RARE, rapid acquisition with relaxation enhancement; SE, spin echo; T1W, T1-weighted; T2W, T2-weighted.

**Table 2:** Pulse sequences used in simulations

	SPGR	RARE	EPI	Spiral
Sequence name	gre.seq	tse.seq	epi.seq	spiral.seq
RF pulse type	sinc	sinc	sinc	Gauss (fat sat), sinc
Num. RF pulse samples (1 us/sample)	3000 (excite)	2500 (excite)	3000 (excite)	8000 (fat sat) 3000 (excite)
		2000 (refocus)		
Num. with-RF	128	153	3	8
Num. with-ADC	128	128	192	4
FOV (mm x mm)	256 × 256	256 x 256	220 × 220	256 × 256
Slice thickness (mm)	3	5	3	3
TR (ms)	12	2000	N/A	N/A
TE (ms)	5	Approx. 100	Approx. 26	Approx. 3
Num. readout samples	128	128	64	13000
Num. phase encodes	128	128, ETL = 16 1 dummy shot	64	N/A
Num. slices	1	1	3	4
Readout sampling ratio	25 us/sample	50 us/sample	4 us/sample	1.6 us/sample
Reconstruction matrix	256 × 256	256 × 256	256 × 256	256 × 256
Oversampling in gridding	2.0	2.0	4.5	2.0

Four different sequences were used for evaluations. These sequences were written in the Pulseq format. ADC, analog to digital converter; EPI, echo planar imaging; ETL, echo train length; N/A, not applicable; RARE, rapid acquisition with relaxation enhancement; SPGR, spoiled gradient echo.

**Table 3:** Number of isochromats and groups, and average processing times using the circles phantom

(a) Number of isochromats and groups				
Total	No grads	Gx only	Gy only	Gz only
20068128	10	2108	2114	480
(b) Processing times				
CPU type	SPGR	RARE	EPI	Spiral
No SIMD	250.34	283.16	317.7	940.94
No SIMD (grouped)	14.91 (63.12)	16.10 (58.44)	5.85 (160.83)	548.65 (1.72)
SIMD	48.51	55.83	53.48	143.6
SIMD (grouped)	12.79 (11.23)	13.96 (10.28)	5.32 (27.00)	72.68 (1.98)

This table contains the simulation results of 4 sequences using the circles phantom. (a) Number of isochromats and groups. While there were 20 million isochromats, the numbers of groups were less than 10000. (b) Average processing times in the simulations. Each ratio of the processing time of the proposed method to that of the conventional method is shown in parentheses. The ratios without SIMD instructions were higher than those with SIMD instructions. CPU, central processing unit; EPI, echo planar imaging; RARE, rapid acquisition with relaxation enhancement; SIMD, single instruction, multiple data; SPGR, spoiled gradient echo.

**Table 4:** Number of isochromats, mean number of groups, and processing times using the brain phantom

(a) Number of isochromats and mean number of groups					
Phantom type	Total	No grads	Gx only	Gy only	Gz only
Ungrouped	284154880	N/A	N/A	N/A	N/A
64 clusters	284154880	64	29904	35112	7795
256 clusters	284154880	253	83460	93242	29317
Ungrouped (+$\Delta B_{0}$)	2273239040	N/A	N/A	N/A	N/A
4096 clusters (+$\Delta B_{0}$)	2273239040	2471.5	160364.2	161403.5	149610.0
16384 clusters (+$\Delta B_{0}$)	2273239040	6927.2	192690.2	193077.5	366271.2
(b) Processing times					
Phantom type	SPGR	RARE	EPI	Spiral	
Ungrouped	678	779.6	737.6	2030.3	
64 clusters	179.5	195.6	75.5	1023.4	
256 clusters	182.5	198.5	79.1	1020.9	
Ungrouped (+$\Delta B_{0}$)	5017.3	5810.0	5526.7	15678.4	
4096 clusters (+$\Delta B_{0}$)	1416.7	1541.7	619.3	8008.4	
16384 clusters (+$\Delta B_{0}$)	1442.9	1569.5	652.2	8046.2	

This table contains the simulation results of 4 sequences using the brain phantom split into partial phantoms. (a) Number of isochromats in the brain phantom, and mean number of groups in the partial phantoms. While the numbers of isochromats were 284 million and 2.3 billion, the numbers of groups in the partial phantoms were less than 1 million. (b) Processing times in the simulations using the SIMD instructions. EPI, echo planar imaging; N/A, not applicable; RARE, rapid acquisition with relaxation enhancement; SPGR, spoiled gradient echo; SIMD, single instruction, multiple data.

## Supplementary Materials

Supplementary figures are available online.
